# Genetic Dissection of Carotenoid Variation by Integrating Quantitative Trait Loci Mapping and Candidate Region Association Study in Sweet Corn

**DOI:** 10.3390/plants15010050

**Published:** 2025-12-23

**Authors:** Yingjie Zhao, Jingtao Qu, Wei Gu, Diansi Yu, Hui Wang, Zhonglin Zhang, Felix San Vicente Garcia, Mengxia Yang, Xiaoyu Sun, Hongjian Zheng, Yuan Guan

**Affiliations:** 1 CIMMYT-China Specialty Maize Research Center, Crop Breeding and Cultivation Research Institute, Shanghai Academy of Agricultural Sciences, Shanghai 201403, China; 2 College of Life Sciences, Shanghai Normal University, Shanghai 200234, China; zhonglin@shnu.edu.cn; 3 International Maize and Wheat Improvement Center (CIMMYT), Texcoco 56237, Mexico

**Keywords:** sweet corn, carotenoids, quantitative trait loci, candidate region association study, GDSL esterase/lipase

## Abstract

Sweet corn is widely cultivated and valued for its palatability and nutritional quality, with kernels accumulating substantial carotenoids, which serve as essential antioxidants and vitamin A precursors. This study elucidated the genetic basis of carotenoid variation in sweet corn kernels by integrating quantitative trait loci (QTL) mapping with a candidate region association study. Seven carotenoid-related traits were quantified in a recombinant inbred line (RIL) population and its parental lines. QTL mapping based on a high-density genotyping-by-target sequencing (GBTS) map and BLUE values across two environments identified 15 loci on chromosomes 5, 6, 7, 8, and 9, explaining 3.83–17.25% of the phenotypic variance. Notably, chromosome 6 harbored a cluster of major-effect QTLs regulating β-cryptoxanthin, zeaxanthin, lutein, total carotenoids, and provitamin A contents. A regional association study within these linkage-defined intervals detected 71 significant SNPs (Bonferroni *p* < 1/n) and identified *Zm00001d036238*, encoding a GDSL esterase/lipase, as a strong candidate gene associated with β-cryptoxanthin accumulation. This gene exhibited kernel-specific expression in the endosperm and harbored a downstream cis-variant (Chr6: 78,466,427) correlated with increased carotenoid content. Allelic effect analysis indicated that the A/A genotype conferred markedly higher β-cryptoxanthin levels than other genotypes. Collectively, these findings provide valuable genetic resources for marker-assisted selection and biofortification breeding to enhance the nutritional quality of sweet corn.

## 1. Introduction

Carotenoids are a class of lipid-soluble isoprenoid pigments widely distributed in plants, responsible for the vivid yellow, orange, and red colors of fruits, vegetables, and floral tissues [1,2]. In plants, carotenoids are integral components of the photosynthetic machinery and serve as precursors for key phytohormones, including abscisic acid and strigolactones, thereby playing vital roles in plant growth, organ development, and abiotic stress adaptation [3,4]. Carotenoids mainly include provitamin A carotenoids (e.g., β-cryptoxanthin, α-carotene, and β-carotene) and non-provitamin A carotenoids (e.g., lutein and zeaxanthin [5,6,7,8]. Sweet corn (*Zea mays* L. *Saccharata*), a fruit-type maize harvested at the milk stage, is widely favored by consumers for its tender texture and sweetness. Its kernels accumulate substantial carotenoids, conferring both nutritional value and commercial appeal. Relative to dent maize, sweet corn represents a genetically distinct maize subpopulation and typically carries endosperm starch-biosynthesis mutations (e.g., *sh2* and *su1*) that redirect carbon flux from starch to sugars [9]. Against this unique high-sugar/low-starch background, favorable alleles identified in dent/field-maize panels [5,10] may not fully extrapolate to sweet corn. Thus, elucidating the genetic basis of carotenoid accumulation in sweet corn can advance our understanding of metabolic and regulatory networks and provide a foundation for biofortification to improve nutritional quality.

The carotenoid biosynthetic pathway begins with geranylgeranyl pyrophosphate (GGPP), synthesized via the methylerythritol phosphate (MEP) pathway [11]. GGPP is condensed into phytoene under the catalysis of phytoene synthase (PSY) and subsequently converted into all-trans-lycopene through a series of desaturation and isomerization reactions catalyzed by phytoene desaturase (PDS), ζ-carotene desaturase (ZDS), and carotenoid isomerase (Z-ISO) [2,11,12]. Lycopene cyclization represents a key branching point of the pathway. Co-action of lycopene ε-cyclase (LCYE) and lycopene β-cyclase (LCYB) yields α-carotene, which is further hydroxylated by β-carotene hydroxylase (HYD) to form lutein, while LCYB alone catalyzes the production of β-carotene, subsequently hydroxylated in two steps by HYD to form β-cryptoxanthin and zeaxanthin [13,14]. Several key biosynthetic genes play pivotal roles in determining carotenoid content in maize kernels. The *psy1* locus underwent a selective sweep during domestication, promoting carotenoid accumulation in the endosperm [15,16]. Natural allelic variation at *LCYE* alters the metabolic flux between the α-carotene and β-carotene branches [17]. Polymorphisms in *crtRB1* (*HYD3*) affect hydroxylation efficiency, with alleles exhibiting reduced transcript levels being associated with elevated β-carotene accumulation [18]. The identification and utilization of such favorable alleles provide valuable genetic resources for biofortification enhancing carotenoid levels in maize kernels. However, despite major advances in characterizing these biosynthetic genes, regulatory mechanisms outside the core pathway, such as carotenoid esterification or sequestration, remain poorly characterized, particularly in sweet corn.

Quantitative trait locus (QTL) mapping and genome-wide association studies (GWAS) have been widely applied to uncover the genetic architecture of complex traits. Linkage mapping based on various biparental populations has revealed that carotenoid variation in maize is typically controlled by a few major-effect QTLs and multiple minor-effect loci [19,20]. Complementarily, GWAS leveraging natural genetic diversity across large maize panels has identified hundreds of single-nucleotide polymorphisms (SNPs) significantly associated with carotenoid components, including novel loci beyond canonical biosynthetic genes [10,21,22,23]. Integrating QTL mapping with GWAS combines the high detection power of linkage analysis with the fine resolution of association mapping, thereby enhancing the identification of strongly associated genetic variants and novel loci underlying complex quantitative traits in multi-parental and meta-analytic studies [5,10].

In order to dissect the genetic architecture of carotenoid accumulation in sweet corn kernels, we investigated carotenoid-related traits in a recombinant inbred line (RIL) population for QTL mapping and an association panel for candidate region association study. This integrated approach identified multiple QTLs, significant SNPs, putative candidate genes and favorable alleles associated with carotenoid variation, thereby providing valuable molecular targets for marker-assisted selection and nutritional biofortification in sweet corn.

## 2. Results

### 2.1. Phenotypic Analysis of Carotenoid-Related Traits in Maize Kernels

**Figure 1:**
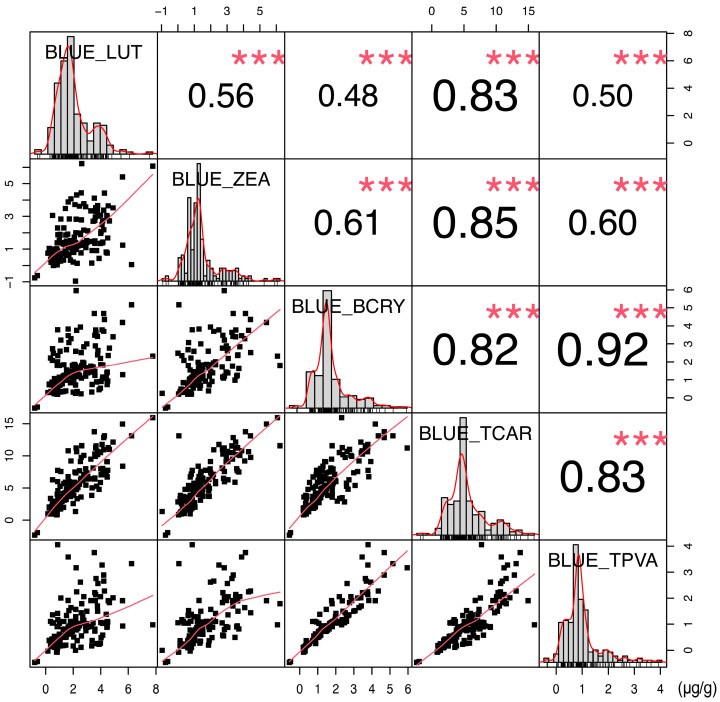
Phenotype distributions and correlations of lutein (LUT), zeaxanthin (ZEA), β-cryptoxanthin (BCRY), total carotenoids (TCAR), and provitamin A (TPVA) in sweet corn RIL population. The diagonal panels display the frequency distribution histograms. The upper triangular panels show the Pearson correlation coefficients (*r*), where asterisks indicate significance at *p* < 0.001 (***). The lower triangular panels present bivariate scatter plots, with black squares representing individual samples and red solid lines indicating linear regression trends.

To elucidate the genetic basis underlying carotenoid variation in sweet corn kernels, seven carotenoid-related traits were quantified in the sweet corn parental lines and a RIL population cultivated under two distinct environments, spring greenhouse (SPG) and summer field (SUF). The analytical panel comprised five fundamental carotenoids, lutein (LUT), zeaxanthin (ZEA), β-cryptoxanthin (BCRY), α-carotene (ACAR), and β-carotene (BCAR), along with two derived metrics, total carotenoids (TCAR) and provitamin A (TPVA). A pronounced contrast was observed between parental lines SHL01 and SHL03. SHL01 exhibited negligible carotenoid accumulation, whereas SHL03 displayed markedly higher levels of LUT (29.32 μg/g), ZEA (16.64 μg/g), BCRY (7.47 μg/g), TCAR (55.23 μg/g), and TPVA (5.43 μg/g), with only trace amounts of ACAR (0.21 μg/g) and BCAR (1.59 μg/g). This parental divergence generated extensive transgressive segregation in the RIL population. Consequently, ACAR and BCAR exhibited low or undetectable concentrations in both environments, with rare extreme values causing overdispersion (CV > 200%) on a zero-inflated background, leaving insufficient variance for QTL mapping and necessitating their exclusion (Table 1). The remaining five traits, LUT, ZEA, BCRY, TCAR, and TPVA, exhibited skewed distributions, implying control by a limited number of major-effect loci (Figure 1 and Figure S1; Table 1). Variance component analysis revealed their modest heritability and substantial environmental influence (Table S1). Strong positive correlations were observed among these traits (*r* = 0.48–0.92; *** *p* < 0.001; Figure 1), further indicating shared regulatory pathways or pleiotropic genetic effects within the carotenoid biosynthetic network.

### 2.2. QTL Mapping of Carotenoid-Related Components

**Figure 2:**
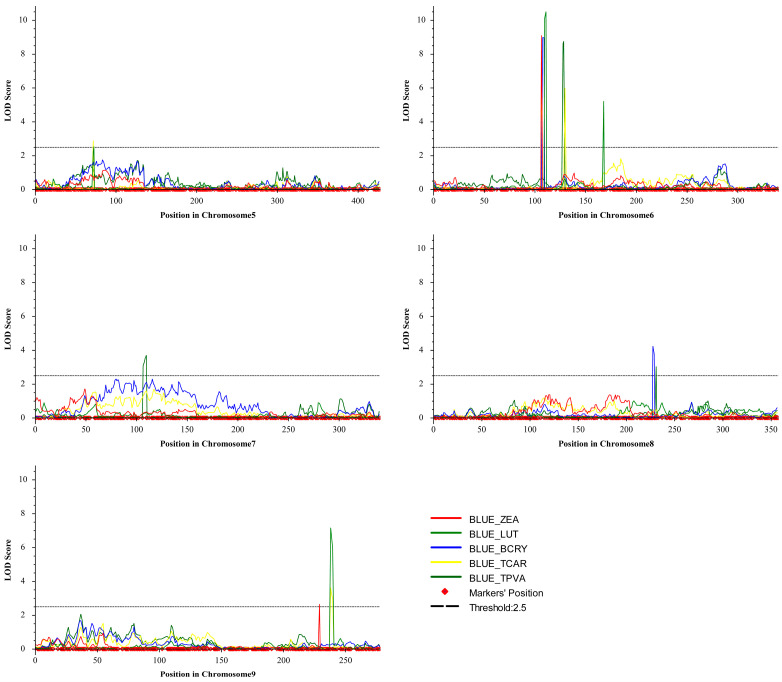
Distributions of QTL intervals for lutein (LUT), zeaxanthin (ZEA), β-cryptoxanthin (BCRY), total carotenoids (TCAR), and provitamin A (TPVA).

**Figure 3:**
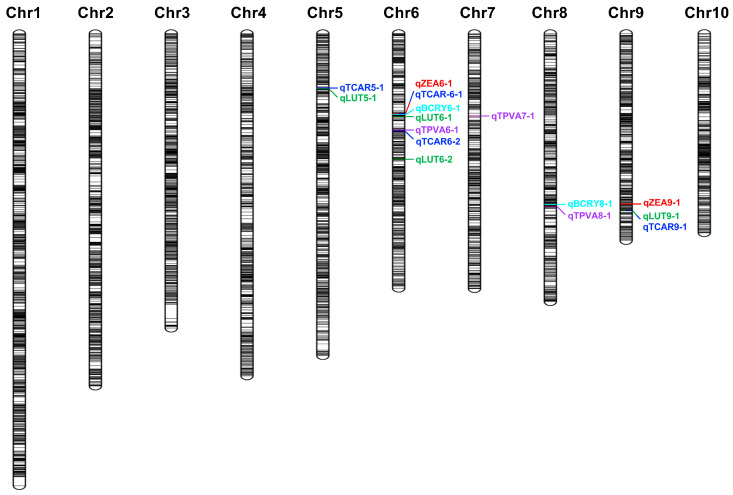
Distributions of identified QTLs for five kernel carotenoid-related traits on genetic linkage maps. QTLs were named as “q + trait abbreviation + chromosome number”.

Genotyping of the RIL population, using the genotyping-by-target sequencing (GBTS) approach based on the SHL01 genome, yielded 5081 high-quality SNP markers to construct a high-density genetic linkage map (unpublished). These markers were evenly distributed across all ten chromosomes, with an average inter-marker distance of 0.71–0.85 cM and no apparent gaps or low-density regions observed. QTL mapping was subsequently performed using the inclusive composite interval mapping (ICIM) method, integrating the high-density linkage map with phenotypic best linear unbiased estimators (BLUEs) derived from two environments. Fifteen loci significantly associated with carotenoid biosynthesis (LOD ≥ 2.5) were detected on chromosomes 5, 6, 7, 8, and 9 (Figure 2 and Figure 3; Table 2). These loci exhibited LOD values ranging from 2.54 to 10.50 and explained 3.83% to 17.25% of the phenotypic variance (PVE), with chromosome 6 harboring the highest QTL density (seven loci, 46.7% of the total).

Among the fifteen loci, seven QTLs with major effects (PVE > 10%) were identified, six of which were concentrated on chromosome 6, indicating that this chromosome serves as the primary genomic region regulating carotenoid accumulation. Within chromosome 6, three consecutive intervals located at 91.76–96.13 Mb, 100.10–100.87 Mb, and 105.71–105.97 Mb were associated with five carotenoid-related traits. The first interval (91.76–96.13 Mb) encompassed *qBCRY6-1* (LOD = 8.97, PVE = 17.04%), which was physically adjacent to two co-localized loci, *qZEA6-1* (LOD = 9.10, PVE = 17.02%) and *qTCAR6-1* (LOD = 6.92, PVE = 14.05%), forming a QTL cluster within this region that may jointly regulate BCRY, ZEA, and TCAR. The second interval (100.10–100.87 Mb) harbored *qLUT6-1* (LOD = 10.50, PVE = 17.25%), which represented the most significant locus influencing LUT content. The third interval (105.71–105.97 Mb) included *qTPVA6-1* (LOD = 8.76, PVE = 16.45%) and *qTCAR6-2* (LOD = 5.98, PVE = 11.97%), which jointly contributed to TPVA and TCAR variation. All chromosome 6 QTLs exhibited positive additive effects (0.29–1.0 µg/g), indicating that alleles from the SHL03 parental line contributed to increased carotenoid accumulation. Furthermore, *qLUT9-1* (LOD = 7.14, PVE = 11.16%) and *qTCAR9-1* (LOD = 3.62, PVE = 7.03%) were identified on chromosome 9, suggesting additional genomic loci influencing carotenoid biosynthesis. The clustering of multiple major QTLs on chromosome 6 highlights this region as a critical genomic hub for carotenoid regulation and a promising target for candidate gene discovery.

### 2.3. Integrating Candidate Region Association Study to Identify Potential Genes

To refine the major QTL intervals and identify potential genes, a regional association study was performed within six QTL regions on chromosome 6 (*qLUT6-1*, *qZEA6-1*, *qBCRY6-1*, *qTCAR6-1*, *qTCAR6-2*, and *qTPVA6-1*). These intervals corresponded to physical positions Chr6: 86,288,907–87,194,754, Chr6: 77,511,836–77,689,404, Chr6: 77,689,405–82,556,250, Chr6: 77,511,836–77,689,404, Chr6: 92,005,442–92,080,125, and Chr6: 91,740,286–92,005,440 bp on the B73 RefGen_v4 reference genome (Table S2), encompassing 5734, 1741, 23,712, 1741, 236, and 585 high-quality SNPs, respectively. Applying a Bonferroni-corrected significance threshold of *p* < 1/n (n representing the number of SNPs per interval), a total of 71 SNPs were identified as significantly associated with carotenoid-related traits (Table S3; Figures S2 and S3). Functional annotation of the significant variants revealed 17 genes with putative biological functions, among which 16 were associated with BCRY and 1 with LUT, while the remaining SNPs were located in intergenic regions or genes of unknown function.

**Figure 4:**
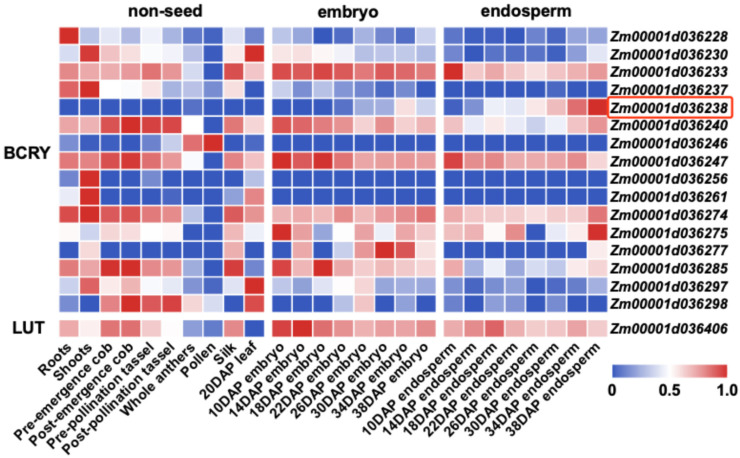
Expression patterns of candidate genes across maize tissues. All tissue samples were collected from the B73 inbred line. Transcript abundance was derived from published RNA-seq datasets [24,25,26]. DAP indicates days after pollination. FPKM values were log_2_-transformed and row-wise normalized to a range of 0–1 using the min-max scaling method to heatmap visualization. The red box marks the candidate gene with a kernel-specific expression pattern.

Integration with 26 publicly available RNA-seq datasets across multiple maize tissues and developmental stages (Table S4) [24,25,26], including root, shoot, leaf, cob, tassel, embryo and endosperm, revealed that *Zm00001d036238* displayed kernel-specific expression, with transcript levels increasing progressively during kernel development and peaking in the endosperm (Figure 4). This spatio-temporal expression pattern is consistent with the primary site of carotenoid accumulation in maize kernels. *Zm00001d036238*, located at Chr6: 78,463,989–78,465,795, encodes a GDSL esterase/lipase belonging to a multifunctional enzyme family involved in lipid metabolism and carotenoid esterification or stabilization in cereals. Accordingly, it was identified as a candidate gene underlying natural variation in BCRY accumulation. Six significant SNPs were detected downstream of this gene (Chr6: 78,466,299; 78,466,427; 78,467,220; 78,576,666; 78,576,946; 78,576,982), with the lead SNP at Chr6: 78,466,427 (*p* = 9.74 × 10^−11^) representing the most significant variant within the *qBCRY6-1* interval.

### 2.4. Allelic Variation and Its Effect on β-Cryptoxanthin Accumulation

**Figure 5:**
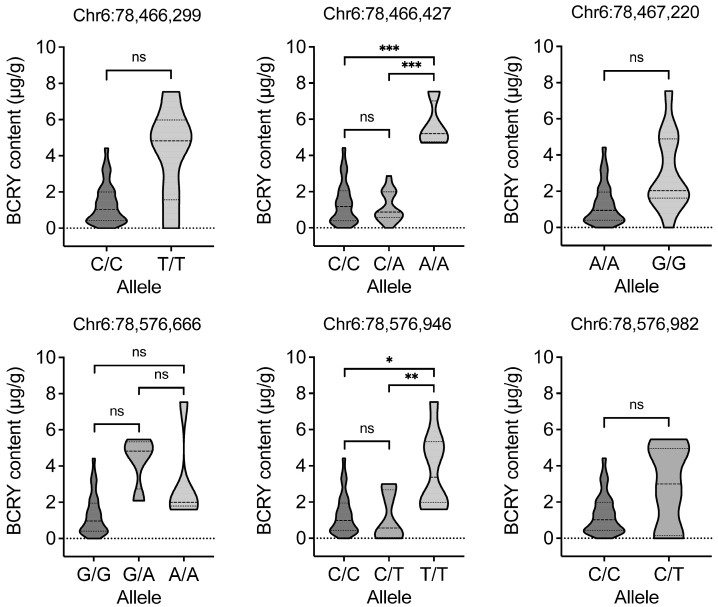
Variation in kernel β-cryptoxanthin (BCRY) content in the sweet corn association panel carrying distinct alleles. The three dashed horizontal lines in violin plots indicate the quartiles. Significant differences are denoted by asterisks using Student’s *t*-test (ns, no significant difference; * *p* < 0.05; ** *p* < 0.01; *** *p* < 0.001).

To characterize the allelic variation surrounding *Zm00001d036238*, local linkage disequilibrium (LD) was analyzed within a ±200 kb window around the lead SNP (Chr6: 78,466,427). Although the six significant SNPs were located in close physical proximity, LD analysis revealed weak inter-marker correlations, and no distinct LD block was observed (Figure S4), suggesting that multiple independent signals may contribute to BCRY variation. Allelic effect analysis demonstrated that the lead SNP (Chr6: 78,466,427) accounted for the largest proportion of phenotypic variance (Figure 5). Highly significant differences in BCRY content were detected among genotypes (*p* < 0.001), with lines carrying the A/A allele (*n* = 4) exhibiting substantially higher BCRY levels than those harboring A/C (*n* = 23) or C/C (*n* = 104) genotypes. This variant, located approximately 1.4 kb downstream of *Zm00001d036238*, is likely to act as a cis-regulatory element modulating carotenoid accumulation.

Collectively, these findings identified *Zm00001d036238* as a priority candidate gene responsible for natural variation in BCRY accumulation and demonstrated the A/A allele at Chr6: 78,466,427 as a favorable variant associated with increased carotenoid deposition. The integration of QTL mapping and candidate region association study effectively refined the genetic architecture of carotenoid accumulation on chromosome 6 and revealed functionally relevant alleles as potential molecular targets for marker-assisted selection, thereby establishing a genetic foundation for biofortification strategies to enhance the nutritional quality of sweet corn.

## 3. Discussion

In this study, 15 QTLs were identified for five carotenoid-related traits, including β-cryptoxanthin, zeaxanthin, lutein, total carotenoids and provitamin A (Figure 2 and Figure 3; Table 2). At least one major-effect QTL (PVE > 10%) was detected for each trait, consistent with previous maize kernel carotenoid QTL studies conducted in diverse genetic backgrounds and population sizes, where a few large-effect QTLs together with several minor-effect loci collectively determine the heritable variation in carotenoid content in biparental populations [19,27,28]. Corresponding intervals in B73 RefGen_v4, four QTLs on chromosomes 6 (*qLUT6-1*, 86,288,907–87,194,754), 8 (*qBCRY8-1*, 145,257,917–146,188,805), and 9 (*qLUT9-1*, *qTCAR9-1*, 154,152,090–154,127,081) (Table S2) were located near key carotenoid biosynthetic genes, including *PSY1* (*Zm00001d036345*), *LCYE* (*Zm00001d011210*), and *HYD5* (*Zm00001d048469*) within 1–3 Mb (Table S5) [10]. Notably, *qLUT6-1* on chromosome 6, the major-effect QTL with the highest PVE (17.25%), was in close proximity to *PSY1*, which has been previously demonstrated as a primary determinant of yellow kernel coloration [16], suggesting that this genomic region consistently influences kernel carotenoid variation across diverse genetic backgrounds.

Overlaying association study onto linkage intervals significantly narrowed candidate regions, thereby improving mapping resolution. Regional association study identified 71 significant SNPs associated with 17 functionally annotated genes, including loci outside the canonical carotenoid biosynthetic pathway (Table S3). Integration with multi-tissue expression data prioritized *Zm00001d036238* as a strong candidate, showing endosperm-specific expression and upregulation during kernel development. *Zm00001d036238* encodes a GDSL esterase/lipase (GELP), a multifunctional enzyme family involved in hydrolyzing diverse lipid substrates and implicated in plant growth and stress responses [29]. Carotenoids can exist in either free or esterified forms; esterification facilitates their sequestration within plant cells and enhances chemical stability, thereby reducing degradation during postharvest storage [3,30,31,32,33]. Notably, the wheat GELP family member XAT-7AD exhibits broad substrate specificity that utilizes multiple acyl donors to esterify lutein, β-cryptoxanthin, and zeaxanthin, thereby enhancing carotenoid stability and retention in grains [34]. Drawing on this functional analogy, it is hypothesized that *Zm00001d036238* might contribute to carotenoid accumulation in sweet corn kernels, potentially by modulating carotenoid esterification or lipid homeostasis. Given the modest sample size, these associations should be interpreted cautiously and validated in larger and/or independent populations. While the precise biochemical activity remains to be experimentally verified, our findings suggest that non-biosynthetic mechanisms, such as esterification, may play an overlooked role in carotenoid homeostasis, warranting further investigation.

Significant SNPs located downstream of *Zm00001d036238*, including the lead SNP at Chr6: 78,466,427 (~1.4 kb from the coding region), indicated that allelic variation was likely driven by cis-regulatory elements. Consistently, a candidate gene association analysis of yellow/orange colored endosperm lines demonstrated that polymorphisms in both the 5’ promoter and 3’ UTR of *LCYE* significantly affect the flux distribution between the α- and β-carotene branches [17]. Moreover, polymorphisms in 5’ TE, Indel4, and 3’ TE region of *crtRB1* modulate hydroxylation efficiency of the encoded enzymes and are significantly associated with β-carotene content and conversion rates in maize kernels [18], with the 3’ TE contributing a two- to ten-fold increase in β-carotene and total provitamin A content [7]. These studies support the crucial role of regulatory variation in controlling kernel carotenoid accumulation. In addition, three indels in the intron, CDS and 3’ UTR of *Zm00001d036238* were detected between ‘SHL01’ and ‘SHL03’ (Table S6), and their functional relevance remains to be determined.

Molecular markers based on functional polymorphisms hold great potential for accelerating the efficient utilization of genetic resources to develop carotenoid-enriched lines. Introgression of favorable *LCYE* and *crtRB1* alleles can elevate provitamin A content in maize kernels to 19.2 μg/g, far exceeding the baseline levels (<2 μg/g) observed in natural germplasm [7,35]. However, these major-effect loci were not detected in our analyses, likely due to limited power (population size and recombination), environmental variance, and potential background-dependent allelic effects in sweet corn. In contrast, allelic effect analysis of the lead SNP Chr6: 78,466,427 revealed that the A/A genotype conferred substantially higher β-cryptoxanthin levels than alternative genotypes, supporting marker-assisted pyramiding of favorable alleles to improve kernel nutritional quality. Collectively, the integration of linkage mapping, candidate region association study, and expression data in this study identified QTLs and candidate genes that deepen our understanding of the genetic architecture underlying carotenoid variation in sweet corn. The GDSL esterase *Zm00001d036238* and its associated polymorphisms provide a complementary target to canonical pathway enzymes, offering valuable genetic resources for biofortification.

## 4. Materials and Methods

### 4.1. Plant Materials and Field Experiments

A RIL population comprising 236 lines was derived from a cross between two sweet corn inbred lines, SHL01 (white-kernel type) and SHL03 (yellow-kernel type). The population was advanced to the F_7_ generation through continuous self-pollination using single-seed descent method. Field experiments were conducted under two environmental conditions, the spring greenhouse (SPG) and the summer field (SUF), at the Zhuanghang Experimental Station of the Shanghai Academy of Agricultural Sciences (Shanghai, China). For the candidate region association study, an association panel of 147 sweet corn inbred lines was used. The germplasm resources were provided by the Shanghai Academy of Agricultural Sciences and planted in a single environment at the seed breeding base in Hainan, China.

All lines were grown in two-row plots, with 11 plants per row. Rows were 2.5 m in length, with 0.6 m spacing between rows and 0.25 m between plants. Well-developed plants were selected for self-pollination. At 21 days after pollination, 15 kernels were collected from the middle part of three healthy ears without visible pest or disease damage, and used for carotenoid content determination.

### 4.2. Carotenoid Quantification

Carotenoids were extracted following the method described by Kurilich and Juvik [36] with slight modifications. Quantification was performed by high-performance liquid chromatography (HPLC) using a reverse-phase YMC C30 CT99S05-2546WT column (5 μm, 250 × 4.6 mm) under isocratic elution at a column temperature of 40 °C and a flow rate of 1 mL/min, with detection set at 450 nm. For quantitative analysis, an external standard calibration curve was constructed using authentic standards of lutein (LUT), zeaxanthin (ZEA), β-cryptoxanthin (BCRY), α-carotene (ACAR), and β-carotene (BCAR). Mixed standard working solutions were prepared at concentrations of 0.5, 1, 2, 5, 8, and 10 μg/mL and filtered through a 0.22 μm membrane prior to injection. Two derived traits were calculated: total carotenoids (TCAR) = ACAR + BCAR + LUT + ZEA + BCRY; and total provitamin A (TPVA) = BCAR + ACAR/2 + BCRY/2.

### 4.3. DNA Extraction and Genotyping

Genomic DNA was extracted from young leaf tissues using a commercial plant DNA extraction kit (Tiangen Biotech, Beijing, China). DNA concentration, quality and integrity were assessed using a Qubit Fluorometer (Thermo Fisher Scientific, Waltham, MA, USA), TGem Spectrophotometer Plus and 1% agarose gel electrophoresis, with all samples exhibiting intact bands, A_260_/A_280_ ratios of ~1.8 and A_260_/A_230_ ratios in the range of 2.0–2.2. Genotyping was performed by MOLBREEDING (Shijiazhuang, China) through GBTS approach of GenoBaits^®^Maize 10K panel [37]. Raw reads were subjected to quality control analysis of adapter removal and read quality trimming using fastp software version 0.23.2 [38] with the parameters: fastp -g -q 5 -u 50 -f 10 -F 10 -n 15 -l 140 --overlap_diff_limit 1 --overlap_diff_percent_limit 10. The clean reads were aligned to SHL01 reference genome (Accession No. GWHFPVK00000000.1; National Genomics Data Center, China National Center for Bioinformation) using bwa software version 0.7.17 [39]. The SNPs were called using GATK4 v4.5.0 and filtered using PLINK v1.9 with the following parameters: missing rate < 20%, heterozygosity < 20%, and minor allele frequency (MAF) > 0.05 [40,41].

### 4.4. QTL Mapping

QTL analysis was performed using the inclusive composite interval mapping (ICIM) method implemented in QTL IciMapping v4.2, with a window size of 5 cM, step size of 1 cM, and 5081 background markers. Phenotypic best linear unbiased estimators (BLUEs) derived across two environments were used as input values. Genetic distances (centiMorgans, cM) were estimated using the Kosambi mapping function. A logarithm of odds (LOD) threshold of 2.5 was applied to declare putative QTL [42]. The proportion of phenotypic variance explained (PVE) was estimated for each locus, and QTLs were named as “q + trait abbreviation + chromosome number”.

To align physical positions across reference assemblies, significant loci anchored to SHL01 genome were cross-referenced to B73 RefGen_v4 (Accession No. GCF_000005005.2; National Center for Biotechnology Information). For each interval, 200-bp flanking sequences were extracted from both sides of its boundaries and aligned to the v4 assembly, and the best-matching position was retained as the corresponding interval (Table S2).

### 4.5. Candidate Region Association Study

Candidate region association study was conducted to identify SNPs associated with carotenoid accumulation in sweet corn kernels. Clean data of the association panel were aligned to B73 RefGen_v4 using bwa software version 0.7.17 with the module of “mem” [39]. SNPs were filtered based on missing data rate, MAF, and genomic position following standard quality control criteria [43]. TASSEL version 5.2.20 was employed to conduct the associate analysis with the Generalized Linear Model (GLM) [44]. Significance threshold was determined using the Bonferroni correction (*p* < 1/n, where n represents the total number of SNPs). Linkage disequilibrium (LD) structure was assessed within a ±200 kb window surrounding the lead SNP [45] and visualized using pairwise r^2^ values with LDBlockShow v1.39 [46]. Haplotype blocks were defined based on the D’ confidence intervals using PLINK v1.9 [40,47].

### 4.6. Statistical Analysis

Descriptive statistics, including skewness, kurtosis, and coefficient of variation (CV), were calculated for all carotenoid-related traits using IBM SPSS Statistics 25.0 (IBM Corp., Armonk, NY, USA). Correlation analyses and trait distribution visualization were performed in R v4.5.1 using the PerformanceAnalytics package. Significant differences were evaluated using Student’s *t*-test. Graphical illustrations were prepared using GraphPad Prism 8 and Microsoft PowerPoint.

## Figures and Tables

**Table 1:** Performance of lutein (LUT), zeaxanthin (ZEA), β-cryptoxanthin (BCRY), α-carotene (ACAR), β-carotene (BCAR), total carotenoids (TCAR) and provitamin A (TPVA) in sweet corn RIL population under two environments.

Environment	Trait	Mean ± SD (μg/g)	Range (μg/g)	Skewness	Kurtosis	CV (%)
SPG	LUT	0.76 ± 0.61	0.11–3.54	2.14	5.48	80.1
	ZEA	0.46 ± 0.67	0.04–5.18	3.97	19	146.38
	BCRY	0.62 ± 0.58	0.02–5.13	4.16	23.1	93.27
	ACAR	0.00	0.00	/	/	/
	BCAR	0.01 ± 0.14	0.00–1.91	12.05	150.76	1065.11
	TCAR	1.85 ± 1.60	0.40–11.13	2.77	8.92	86.28
	TPVA	0.32 ± 0.35	0.01–3.05	4.71	27.69	108.2
SUF	LUT	3.21 ± 2.55	0.31–8.92	0.69	−0.91	79.46
	ZEA	2.51 ± 2.18	0.00–8.65	1.07	−0.05	86.85
	BCRY	2.35 ± 2.09	0.04–8.25	0.97	−0.16	88.95
	ACAR	0.10 ± 0.43	0.00–3.32	5.15	29.23	420.62
	BCAR	0.33 ± 0.71	0.00–4.38	2.88	9.81	215.78
	TCAR	8.50 ± 6.16	0.85–25.00	0.81	−0.41	72.49
	TPVA	1.55 ± 1.56	0.02–7.93	1.42	1.77	100.8

**Table 2:** QTL intervals of five kernel carotenoid-related traits. Positive (+) or negative (−) additive effects indicated that the SHL03 or SHL01 allele increased trait expression, respectively.

Trait	QTL	Chromosome	Marker Interval	LOD	PVE (%)	Additive
LUT	*qLUT5-1*	5	14,745,795–15,981,919	2.54	3.83	0.25
	*qLUT6-1*	6	100,095,064–100,870,497	10.50	17.25	0.52
	*qLUT6-2*	6	121,751,651–121,788,281	5.22	8.24	0.36
	*qLUT9-1*	9	161,970,440–161,911,412	7.14	11.16	0.42
ZEA	*qZEA6-1*	6	91,756,773–91,927,299	9.10	17.02	0.48
	*qZEA9-1*	9	158,823,760–158,813,271	2.65	4.70	0.25
BCRY	*qBCRY6-1*	6	91,927,299–96,134,979	8.97	17.04	0.44
	*qBCRY8-1*	8	151,902,915–152,790,422	4.23	7.55	−0.29
TCAR	*qTCAR5-1*	5	15,584,794–14,745,795	2.89	5.60	0.63
	*qTCAR6-1*	6	91,756,773–91,927,299	6.92	14.05	1.00
	*qTCAR6-2*	6	105,941,426–105,969,916	5.98	11.97	0.92
	*qTCAR9-1*	9	161,970,440–161,911,412	3.62	7.03	0.71
TPRA	*qTPVA6-1*	6	105,711,040–105,941,426	8.76	16.45	0.29
	*qTPVA7-1*	7	82,417,620–82,290,864	3.68	6.55	−0.18
	*qTPVA8-1*	8	155,431,072–157,945,924	3.03	5.43	−0.17

## Data Availability

The original contributions presented in this study are included in the article. Further inquiries can be directed to the corresponding authors.

## Supplementary Materials

The following supporting information can be downloaded at: https://www.mdpi.com/article/10.3390/plants15010050/s1, Figure S1: Distribution and normality assessment for carotenoid-related traits; Figure S2: Principal component analysis of the sweet corn association panel; Figure S3: Manhattan and Q-Q plots for candidate region association study; Figure S4: Linkage disequilibrium and haplotype block structure of the *qBCRY6-1* locus; Table S1: Variance component analysis for carotenoids-related traits across two environments; Table S2: Summary of QTL intervals for carotenoid-related traits from the SHL01 assembly and their converted coordinates in the B73 RefGen_v4 genome; Table S3: Significant SNPs and candidate genes associated with carotenoid-related traits in sweet corn kernels; Table S4: List of published RNA-Seq data used to find kernel-specific genes; Table S5: List of genes in the carotenoid biosynthesis pathway; Table S6: Sequence polymorphisms in *Zm00001d036238* between SHL01 and SHL03.
