# Supplementary material for: Genetic Dissection of Carotenoid Variation by Integrating Quantitative Trait Loci Mapping and Candidate Region Association Study in Sweet Corn
Source: Plants (Basel). 2025 Dec 23;15(1):50. doi: 10.3390/plants15010050 (PMC12787729; doi:10.3390/plants15010050)
Supplement: Supplementary file 1 [file plants-15-00050-s001.zip › Supplementary Files/Supplementary Figures.doc]

**Figure S1 Distribution and normality assessment for carotenoid-related traits.**

Histograms (left) showed the empirical distributions of best linear unbiased estimators (BLUEs) for lutein (LUT), zeaxanthin (ZEA), β-cryptoxanthin (BCRY), total carotenoids (TCAR), and provita-min A (TPVA). Quantile-quantile (Q-Q) plots (right) compared sample quantiles (x-axis) with theoretical quantiles under a normal distribution (y-axis). Furthermore, Shapiro-Wilk normality tests were conducted using the shapiro.test() function in R v4.5.1, revealing p-values of 4.422×10⁻¹⁰ for LUT, 9.692×10⁻¹³ for ZEA, 5.196×10⁻¹³ for BCRY, 2.754×10⁻⁹ for TCAR, and 6.441×10⁻¹⁴ for TPVA, which indicated that none of the traits fully conform to a normal distribution.

**Figure S2 Principal component analysis of the sweet corn association panel.**

**Figure S3 Manhattan and Q-Q plots for candidate region association study.**

**Figure S4 Linkage disequilibrium and haplotype block structure of the *qBCRY6-1* locus.**

The color of each cell within the plot indicates the r² value, from white (r² = 0) to dark red (r² = 1).
